# Enhanced Tumor-to-Background Contrast with [^52^Mn]Mn-BPPA-Bevacizumab VEGF-Targeted Immuno-PET in Cervical Cancer

**DOI:** 10.3390/ph19030517

**Published:** 2026-03-22

**Authors:** Csaba Csikos, Minh Toàn Ngô, Adrienn Vágner, Gábor Nagy, Gábor Ország, Tamás Nagy, Balázs Váradi, Gergő Zoltán Sajtos, István Kapus, Zoltán Szoboszlai, Dezső Szikra, Gyula Tircsó, Zoárd Tibor Krasznai, Szabolcs Molnár, Ildikó Garai, György Trencsényi

**Affiliations:** 1 Division of Nuclear Medicine and Translational Imaging, Department of Medical Imaging, Faculty of Medicine, University of Debrecen, H-4032 Debrecen, Hungary; 2 Gyula Petrányi Doctoral School of Clinical Immunology and Allergology, Faculty of Medicine, University of Debrecen, H-4032 Debrecen, Hungary; 3 Scanomed Ltd., H-4032 Debrecen, Hungary; 4 Department of Physical Chemistry, Institute of Chemistry, Faculty of Science and Technology, University of Debrecen, H-4032 Debrecen, Hungarygyula.tircso@science.unideb.hu (G.T.); 5 Doctoral School of Chemistry, Faculty of Science and Technology, University of Debrecen, H-4032 Debrecen, Hungary; 6 Department of Obstetrics and Gynaecology, Faculty of Medicine, University of Debrecen, H-4032 Debrecen, Hungarymolnar.szabolcs@med.unideb.hu (S.M.)

**Keywords:** ^52^Mn, chelator, BPPA, bevacizumab, cervical cancer, immuno-PET, PET/MRI, VEGF

## Abstract

**Background/Objectives**: Radiolabeled bevacizumab-based immuno-PET tracers enable a non-invasive quantification of VEGF-A expression in gynecologic malignancies. While the previously reported [^52^Mn]Mn-DOTAGA-bevacizumab demonstrated selective VEGF-A-targeted uptake in a KB-3-1 cervix carcinoma mouse model, further improvements in chelator stability and tumor-to-background contrast remain desirable. The recently developed BPPA chelator exhibits exceptionally high Mn(II) complex stability and favorable radiolabeling characteristics. This study aimed to characterize the in vivo biodistribution of [^52^Mn]Mn-BPPA-bevacizumab, and to compare the tumor-to-background ratios of [^52^Mn]Mn-BPPA-bevacizumab with the previously published values of [^52^Mn]Mn-DOTAGA-bevacizumab in VEGF-A-expressing cervix carcinoma. **Methods**: Female KB-3-1 tumor-bearing CB17 SCID mice underwent PET/MRI imaging following intravenous administration of [^52^Mn]Mn-BPPA-bevacizumab. SUV_mean_ values were measured in various organs and in the subcutaneously injected tumor, and tumor-to-organ ratios were calculated at various time points up to 10 days post-injection. **Results**: [^52^Mn]Mn-BPPA-bevacizumab demonstrated sustained tumor uptake, with tumor SUVmean values increasing from approximately 1.0 at 4 h to peak values of approximately 2.4–2.5 at 72 h post-injection. Tumor-to-background ratios increased progressively over time and were significantly higher for [^52^Mn]Mn-BPPA-bevacizumab compared with previously reported [52Mn]Mn-DOTAGA-bevacizumab, particularly for tumor-to-blood, tumor-to-liver and tumor-to-lung ratios at later imaging time points (*p* < 0.0001). **Conclusions**: The novel [^52^Mn]Mn-BPPA-bevacizumab tracer exhibits satisfactory in vitro and in vivo stability for PET imaging, high VEGF-A-specific tumor uptake, and markedly improved tumor-to-background ratios compared to the previously published DOTAGA-based probe. These results position [^52^Mn]Mn-BPPA-bevacizumab as a highly promising next-generation immuno-PET agent for imaging VEGF-A-expressing gynecologic malignancies and for guiding anti-angiogenic therapies.

## 1. Introduction

Angiogenesis plays a central role in the growth, progression, and metastatic spread of gynecologic malignancies, including cervical carcinoma [1,2]. Vascular endothelial growth factor A (VEGF-A) is a secreted dimeric glycoprotein that functions as a principal ligand driving tumor angiogenesis, and its overexpression has been linked to poor prognosis, increased tumor aggressiveness, and reduced overall survival in cervical cancer [3]. As a result, anti-angiogenic therapy with bevacizumab, an FDA-approved monoclonal antibody targeting VEGF-A, has become an important component of the treatment algorithm for advanced, recurrent, and metastatic disease [4,5,6]. Despite its therapeutic value, no clinically accepted biomarker exists to non-invasively quantify VEGF-A expression or predict which patients are most likely to benefit from bevacizumab therapy.

Although circulating VEGF levels can be quantified using blood-based assays such as ELISA, these measurements do not necessarily reflect the spatial distribution of VEGF expression within tumors or across metastatic sites. Likewise, tissue-based methods such as immunohistochemistry or mRNA analysis are inherently limited by sampling bias, as they evaluate only a small portion of the tumor and therefore cannot capture the full extent of intratumoral heterogeneity or the overall tumor burden [7]. In contrast, immuno-PET imaging enables whole-body, non-invasive visualization and quantification of VEGF expression in vivo, allowing assessment of the entire disease burden. Furthermore, labeling the therapeutic antibody itself, such as bevacizumab, provides the opportunity to directly image the pharmacological target of therapy, potentially enabling more precise identification of patients whose tumors exhibit sufficient VEGF availability to benefit from anti-angiogenic treatment. Importantly, because immuno-PET imaging can be repeated longitudinally without the need for invasive tissue sampling, it may also allow non-invasive monitoring of dynamic changes in VEGF expression during treatment, thereby facilitating early detection of emerging therapeutic resistance [8,9].

However, antibody-based PET tracers require radioactive metal ions with long physical half-lives that match the biological kinetics of immunoglobulins [10,11]. Manganese-52 (^52^Mn), with its 5.59-day half-life, favorable positron energy spectrum and chemical properties that enable facile complexation, represents an attractive isotope for immuno-PET applications [12,13]. Metal ions, however, cannot be conjugated directly to antibodies, only through chelators [14]. Our previous work employing [^52^Mn]Mn-DOTAGA-bevacizumab in a KB-3-1 cervix carcinoma mouse model demonstrated good tumor specificity and sustained uptake, confirming VEGF-A-targeted imaging feasibility [15].

While DOTAGA is a well-established chelator for metal-based radiopharmaceuticals [16], its Mn(II) complexes exhibit moderate kinetic inertness, and further enhancement of complex stability may improve tumor-to-background contrast in antibody imaging. The newly developed bis-pyclen picolinate (BPPA) chelator represents a next-generation chelating agent designed to bind Mn(II) with exceptionally high thermodynamic stability and favorable labeling characteristics under mild, antibody-compatible conditions [17]. Preclinical evaluation of BPPA-conjugated trastuzumab has demonstrated superior radiochemical performance, prolonged in vivo stability, and significantly enhanced tumor-to-background ratios compared to traditional DOTA-based systems [18]. This highlights the importance of choosing an optimal chelator as it can influence the in vivo behavior of the radiopharmaceutical. These findings suggest that ^52^Mn-BPPA-based immuno-PET tracers could offer substantial advantages for VEGF-A imaging as well.

**Figure 1:**
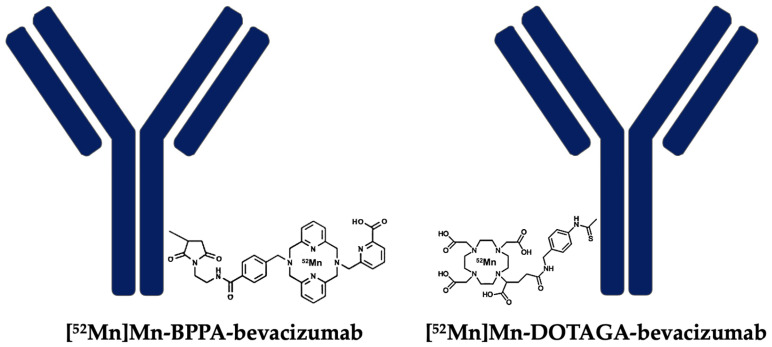
Chemical structures of the bifunctional chelators used for antibody conjugation and radiolabeling with manganese-52.

In light of these developments, the present study aimed to characterize the in vivo biodistribution of [^52^Mn]Mn-BPPA-bevacizumab and compare the tumor-targeting performance and tumor-to-background ratios of the BPPA-based tracer to the previously published [^52^Mn]Mn-DOTAGA-bevacizumab (Figure 1) [15]. We hypothesized that the higher stability of the Mn(II)–BPPA complex would translate into improved imaging contrast, lower off-target activity, and superior tumor visualization in VEGF-A-expressing cervical carcinoma xenografts.

## 2. Results

### 2.1. Radiolabeling and In Vitro Stability of [^52^Mn]Mn-BPPA-Bevacizumab

The successful conjugation of the BPPA-Bu-pMMA chelator to bevacizumab was confirmed by mass spectrometry (MS) analysis (Figure S1). The MS spectrum of the native bevacizumab showed a base peak at 149,198 Da, corresponding to the intact monoclonal antibody. Following the conjugation reaction with a 20-fold molar excess of the chelator, a characteristic shift towards higher molecular weights was observed in the mass spectrum. Deconvolution of the MS spectra revealed a distribution of species with varying chelator-to-antibody ratios for the BPPA-bevacizumab conjugate. The MS analysis of the BPPA-bevacizumab conjugate revealed a distribution of species with different chelator-to-antibody ratios. The primary peaks were identified at 149,150 Da (unmodified antibody, 42% relative abundance), 149,812 Da (1 chelator per antibody, 33%), 150,474 Da (2 chelators per antibody, 17%), and 151,165 Da (3 chelators per antibody, 8.3%). These results demonstrate the formation of a stable conjugate with an average chelator-to-antibody ratio of 0.92. The presence of multiple chelators per protein molecule ensures sufficient binding sites for the ^52^Mn isotope while maintaining the structural integrity of the antibody.

After labeling with ^52^Mn, the radiochemical purity of the final product was determined to be 88% by radio-HPLC and 92% by radio-TLC. Based on the HPLC chromatogram, the fraction of unlabeled (free) isotope was 10%. The specific activity of the labeled conjugate was 39.1 MBq/mg at the time of synthesis.

Serum stability measurements revealed substantial instability; the RCP decreased to 69% on Day 1, followed by total degradation (0% RCP) by Day 4 (Figure S2).

The log *p* value for the ^52^Mn-BPPA-bevacizumab was determined to be −2.24 ± 0.11. This negative value indicates the hydrophilic nature of the radiolabeled antibody conjugate, which is typical for large protein-based complexes.

### 2.2. In Vivo Biodistribution of [^52^Mn]Mn-BPPA-Bevacizumab

Most of [^52^Mn]Mn-BPPA had already been excreted by the time our measurements started (4 h post-injection), and SUV_mean_ values of the measured organs were consistently below 0.5 across all time points (Table S1). This rapid elimination is consistent with the high in vivo stability of the Mn-BPPA complex. If substantial demetallation had occurred, the released ^52^Mn would be expected to follow the biodistribution pattern of free manganese, characterized by accumulation in organs such as the pancreas, salivary glands, and kidneys, rather than undergoing rapid renal clearance.

**Figure 2:**
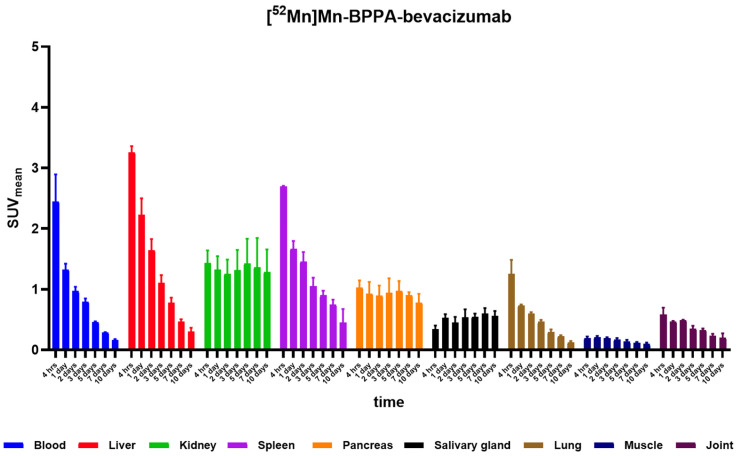
Biodistribution of [^52^Mn]Mn-BPPA-bevacizumab. SUV_mean_ values in blood and selected organs (liver, kidney, spleen, pancreas, salivary gland, lung, muscle, and joint) at 4 h, 1, 2, 3, 5, 7, and 10 days post-injection, as determined by longitudinal PET/MRI imaging. Data are presented as mean ± SD and obtained from *n* = 5 animals. The tracer shows high initial blood pool and hepatic activity followed by progressive clearance from non-target tissues, while uptake in muscle and joint remains low throughout the imaging period.

In contrast, the elimination of [^52^Mn]Mn-BPPA-bevacizumab was noticeably slower, reinforcing the satisfactory in vivo stability of [^52^Mn]Mn-BPPA-bevacizumab. High initial blood pool activity was observed at 4 h post-injection, followed by a rapid and continuous clearance over time, resulting in low blood SUV_mean_ values by days 5–10. Hepatic uptake was prominent at early time points, with the highest liver activity detected at 4 h, followed by a marked decline during the first 48–72 h and further gradual washout thereafter. Renal uptake was moderate and relatively stable throughout the imaging period, showing only minor temporal variation. Uptake in the spleen was initially high, peaking at early time points, and subsequently decreased in a time-dependent manner. Activity in the pancreas and salivary glands remained moderate and relatively constant across all time points, while lung uptake showed an early peak followed by rapid clearance. Muscle and joint uptake remained low throughout the study, demonstrating minimal nonspecific accumulation (Figure 2 and Table S2).

Overall, [^52^Mn]Mn-BPPA-bevacizumab exhibited progressive clearance from non-target tissues, resulting in steadily improving tumor-to-background contrast over time.

### 2.3. Tumor Uptake and Time–Activity Characteristics

**Figure 3:**
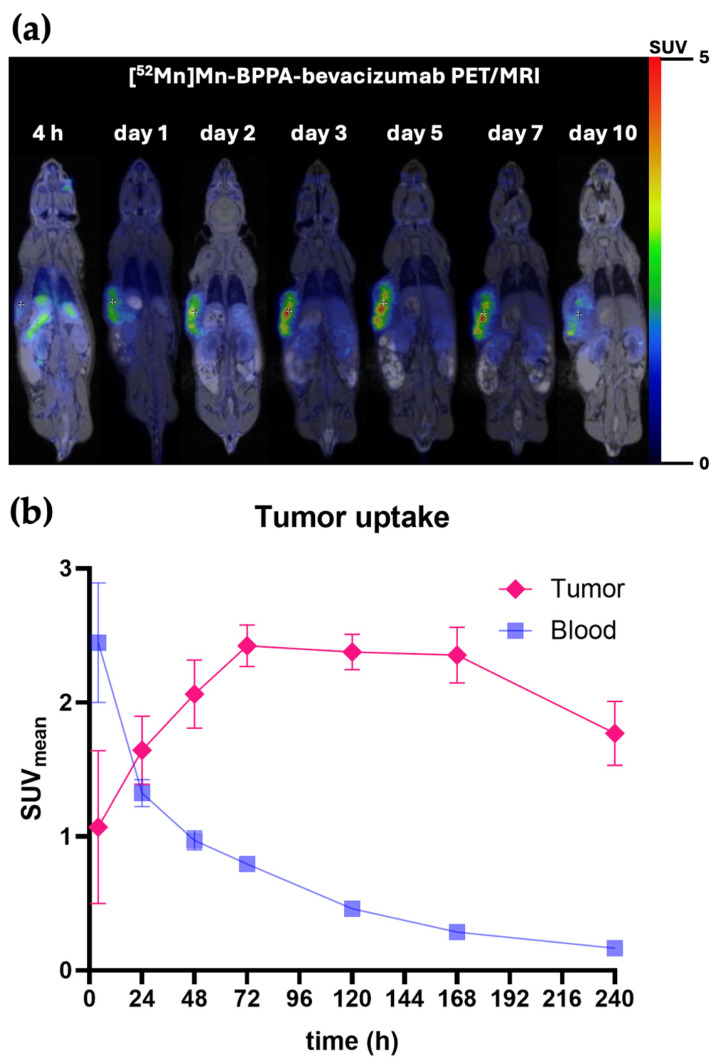
Tumor uptake of [^52^Mn]Mn-BPPA-bevacizumab in KB-3-1 cervix carcinoma xenografts. (**a**) Representative attenuation-corrected coronal PET/MRI images of KB-3-1 cervix carcinoma-bearing mice acquired at multiple time points following intravenous administration of [^52^Mn]Mn-BPPA-bevacizumab. Tumors are clearly visualized from early imaging time points, with progressively improving contrast at later time points as background activity decreases. (**b**) Time–activity curve showing tumor uptake of [^52^Mn]Mn-BPPA-bevacizumab expressed as SUV_mean_ values from 4 h to 240 h post-injection. Tumor uptake increases during the first 72 h, reaches a plateau between days 3 and 7, and shows a moderate decline by day 10, indicating sustained tumor retention. Data are presented as mean ± SD and obtained from *n* = 5 animals/time point.

Tumor uptake of [^52^Mn]Mn-BPPA-bevacizumab increased progressively during the first 72 h post-injection, reaching a plateau between days 3 and 7 (Figure 3b and Table S2). Tumor SUV_mean_ values increased from approximately 1.0 at 4 h to peak values of approximately 2.4–2.5 at 72 h. After reaching this plateau, tumor uptake remained stable up to day 7 and showed a moderate decline by day 10, while remaining clearly distinguishable from background tissues (Figure 3a).

The time–activity curve demonstrates sustained tumor retention with slow washout kinetics, consistent with specific VEGF-A-mediated binding of the BPPA-based conjugate. Although a more sustained and slightly higher average tumor uptake was observed with [^52^Mn]Mn-BPPA-bevacizumab compared with the previously reported [^52^Mn]Mn-DOTAGA-bevacizumab [15], the difference in absolute tumor uptake did not reach statistical significance at any measured time point. This may be partly attributable to the relatively large SD observed in the DOTAGA-based tracer measurements [15].

### 2.4. Comparison of Tumor-to-Background Ratios: BPPA vs. DOTAGA

**Figure 4:**
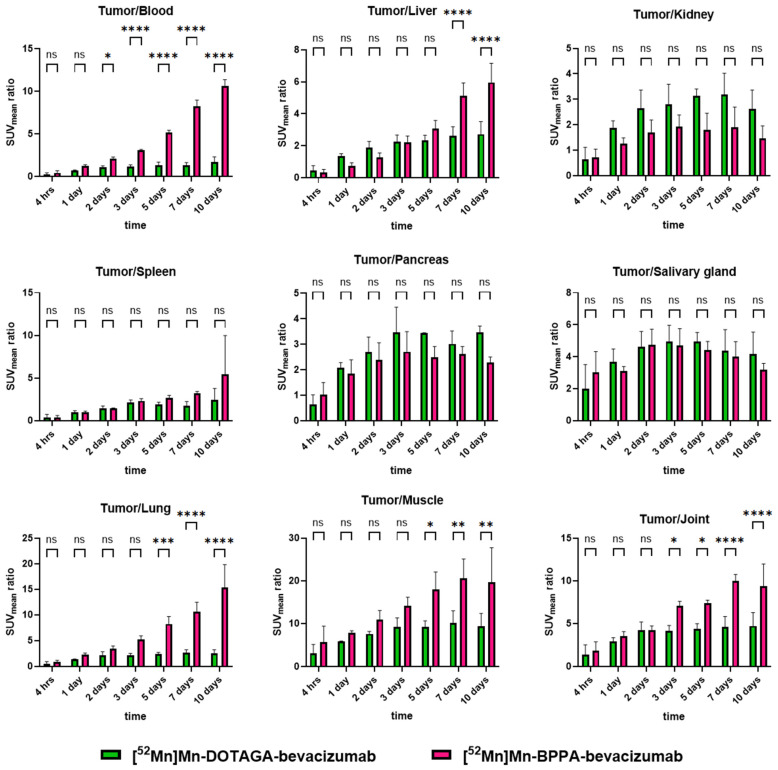
Quantitative comparison of tumor-to-background ratios for [^52^Mn]Mn-BPPA-bevacizumab and [^52^Mn]Mn-DOTAGA-bevacizumab in KB-3-1 cervix carcinoma xenografts. Tumor-to-blood, tumor-to-liver, tumor-to-kidney, tumor-to-spleen, tumor-to-pancreas, tumor-to-salivary gland, tumor-to-lung, tumor-to-muscle, and tumor-to-joint ratios were calculated using SUV_mean_ values at 4 h, 1, 2, 3, 5, 7, and 10 days post-injection. [^52^Mn]Mn-BPPA-bevacizumab demonstrates significantly higher tumor-to-blood, tumor-to-liver, tumor-to-lung, tumor-to-muscle, and tumor-to-joint ratios at late imaging time points, while no significant differences are observed for kidney-, spleen-, pancreas-, or salivary gland-based ratios. Data are shown as mean ± SD, obtained from *n* = 5 animals/time points/radiopharmaceutical. Statistical significance is indicated as ns (not significant), * *p* < 0.05, ** *p* < 0.01, *** *p* < 0.001, **** *p* < 0.0001.

Tumor-to-background ratios of [^52^Mn]Mn-BPPA-bevacizumab were directly compared with previously published values obtained using [^52^Mn]Mn-DOTAGA-bevacizumab under identical experimental conditions [15]. Tumor-to-background ratios are shown in Figure 4.

Tumor-to-blood ratios increased significantly over time for both tracers; however, [^52^Mn]Mn-BPPA-bevacizumab demonstrated significantly higher ratios from day 2 onward, with the difference becoming highly significant at days 3, 5, 7, and 10 (*p* < 0.0001). A similar pattern was observed for tumor-to-liver ratios, where BPPA-based conjugates exhibited markedly superior contrast at late imaging time points, particularly at days 7 and 10.

Tumor-to-lung ratios showed a pronounced and statistically significant advantage for [^52^Mn]Mn-BPPA-bevacizumab at later time points, reflecting faster pulmonary clearance. Tumor-to-muscle and tumor-to-joint ratios increased continuously throughout the study, reaching very high values at days 7 and 10, with significant superiority of the BPPA-based tracer compared to DOTAGA.

Tumor-to-kidney, tumor-to-spleen, tumor-to-pancreas, and tumor-to-salivary gland ratios showed no significant differences between the two tracers across measured time points. These findings indicate that the improved contrast achieved with the BPPA chelator is primarily driven by enhanced tumor retention and faster clearance from blood and non-target soft tissues, rather than altered uptake in excretory or physiologically active organs.

### 2.5. Confirmation of VEGF-A Expression

**Figure 5:**
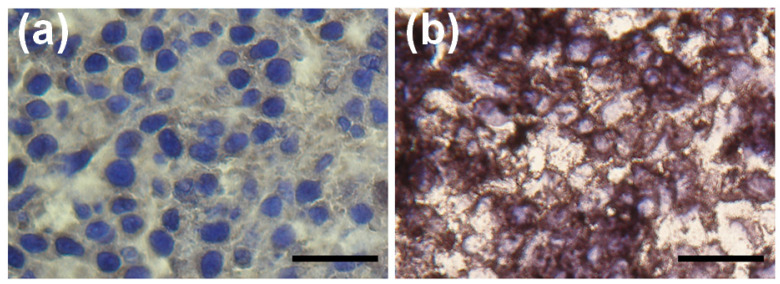
Immunohistopathological analysis of KB-3-1 tumor xenografts. Microscopic images of negative control staining (**a**) and anti-VEGF mAb-DAB immunostaining (**b**) of xenograft tumor sections. Bar: 50 μm; magnification ×200.

Histological analysis performed on excised tumor sections at the end of the imaging experiments confirmed strong VEGF expression in the KB-3-1 xenografts. Tumor tissues exhibited intense staining (Figure 5).

## 3. Discussion

Immuno-PET enables not only highly sensitive tumor detection but also specific, non-invasive visualization of molecular pathophysiological processes in vivo [19]. By exploiting the target selectivity of monoclonal antibodies, immuno-PET allows whole-body assessment of biomarker expression and overcomes key limitations of histopathology-based methods, such as sampling bias and intratumoral heterogeneity [9,20].

In angiogenesis-driven malignancies, including cervical carcinoma, imaging of VEGF-A expression is therefore of particular clinical interest [21]. Although imaging of membrane-bound receptors is common in molecular imaging, targeting VEGF-A itself offers several advantages in the context of angiogenesis-driven tumors [21]. VEGF-A is produced and secreted by tumor cells and represents a key driver of tumor angiogenesis, making it a biologically relevant marker of the angiogenic tumor microenvironment. Imaging VEGF-A therefore enables the assessment of the ligand responsible for activating the VEGF signaling pathway rather than only the downstream VEGFR expression on endothelial cells [22]. Furthermore, bevacizumab is a clinically approved therapeutic antibody that directly targets VEGF-A [23]. Therefore, radiolabeling the therapeutic antibody itself allows visualization of the drug–target interaction in vivo and may provide valuable information for predicting response to anti-angiogenic therapy.

A fundamental requirement for successful immuno-PET is the use of radionuclides whose physical half-life matches the slow pharmacokinetics of immunoglobulins. Full-length antibodies typically require several days to achieve optimal tumor accumulation and sufficient background clearance, making long-lived positron emitters essential [24]. Zirconium-89 (^89^Zr) has become the most widely used radionuclide for immuno-PET due to its suitable half-life (t_1/2_ = 3.27 days); however, its limited in vivo chelation stability can result in radiometal release and nonspecific bone accumulation, which complicates image interpretation and raises dosimetry concerns [25,26,27].

^52^Mn represents a promising alternative for antibody-based PET imaging. Its physical half-life (t_1/2_ = 5.59 days) is well matched to antibody kinetics; its relatively low positron energy (242 keV) allows high-resolution imaging [12,13]. Furthermore, the coordination chemistry of manganese is well characterized thanks to its use as an MRI contrast agent. Nevertheless, there have only been a handful of articles published with ^52^Mn-labeled antibodies, most of them evaluating breast cancer and HER2 status [15,18,28,29,30,31].

When paired with appropriate chelators, ^52^Mn can form stable complexes that minimize in vivo demetallation. In this regard, the BPPA chelator offers high thermodynamic stability and favorable labeling properties under mild conditions compatible with monoclonal antibodies [18].

Building directly on our previously published work with [^52^Mn]Mn-DOTAGA-bevacizumab, which demonstrated selective VEGF-A-targeted tumor accumulation in the same KB-3-1 cervical cancer model, the present study was designed to address limitations related to chelator performance. While DOTAGA enabled stable antibody labeling and clear tumor visualization, its moderate Mn(II) kinetic inertness and limited achievable specific activity may constrain tumor-to-background contrast at later imaging time points.

BPPA chelator was therefore evaluated as a next-generation Mn(II) ligand with the aim of improving in vivo imaging contrast through optimized coordination chemistry. In the present study, [^52^Mn]Mn-BPPA-bevacizumab demonstrated sustained tumor uptake and significantly improved tumor-to-background ratios compared to the previously reported DOTAGA-based analogue, while preserving VEGF-A-specific tumor targeting. Importantly, the improved imaging performance of [^52^Mn]Mn-BPPA-bevacizumab was not driven by a disproportionate increase in absolute tumor uptake as SUV_mean_ values of approximately 2.5 are considered to be moderate, but rather by a more favorable balance between sustained tumor retention and accelerated clearance from blood and non-target tissues. This distinction is critical, as tumor-to-background contrast, not absolute SUV alone, ultimately determines lesion detectability and quantitative reliability in immuno-PET imaging. These findings extend our earlier work and confirm that BPPA-based chelation improves the in vivo imaging characteristics of ^52^Mn-labeled antibodies.

The superior tumor-to-background ratios observed with the BPPA-based tracer can be attributed to the high thermodynamic stability and kinetic inertness of the Mn(II)–BPPA complex. This robust coordination allows efficient radiolabeling under mild, antibody-compatible conditions and enables higher effective specific activity compared with DOTAGA-based conjugates. As a consequence, a greater fraction of administered antibody molecules contributes to the PET signal, while nonspecific background activity decreases more rapidly, resulting in earlier and more pronounced contrast differentiation between VEGF-A-positive tumors and surrounding tissues.

In addition, the relatively low chelator-to-antibody ratio achieved with BPPA conjugation reflects limited chemical modification of the antibody. Prior studies have suggested that increasing chelator density may influence immunoreactivity and pharmacokinetics through alterations in physicochemical properties such as charge and hydrophilicity; however, these effects appear to be modest and highly dependent on the chelator type and conjugation strategy [32]. In the present study, the improved in vivo performance of the BPPA-based tracer is therefore more likely to be attributed to efficient Mn(II) coordination and higher effective molar activity, rather than to differences in antibody modification or target-binding properties.

Notably, tumor-to-kidney, tumor-to-pancreas, and tumor-to-salivary gland ratios did not differ significantly between BPPA- and DOTAGA-based tracers, indicating preserved physiological handling in classical excretory and secretory organs. In contrast, reduced hepatic activity observed with the BPPA-based tracer at late time points likely reflects faster clearance of circulating antibody-associated activity from the liver blood pool rather than altered hepatobiliary excretion.

The radiochemical purity of the final [^52^Mn]Mn-BPPA-bevacizumab preparation was 88% by radio-HPLC and 92% by radio-TLC. The residual activity was predominantly present as [^52^Mn]Mn-BPPA, and additional purification attempts did not improve the radiochemical purity, suggesting the presence of a reversible equilibrium associated with the BPPA-Bn-pMMA conjugation chemistry. Serum stability studies demonstrated progressive degradation of the radiolabeled conjugate in vitro, with the radiochemical purity decreasing from 69% at day 1 to 0% by day 4. This species most likely corresponds to [^52^Mn]Mn-BPPA, suggesting that the reduced in vitro stability may primarily reflect instability at the linker–antibody interface rather than dissociation of the [^52^Mn]Mn-BPPA complex itself. Consistent with this interpretation, biodistribution studies showed rapid clearance of small radiometal complexes from the circulation, indicating that released [^52^Mn]Mn-BPPA is efficiently eliminated and therefore unlikely to substantially compromise imaging contrast. Moreover, the persistent tumor uptake observed at later imaging time points suggests that the in vivo stability of the tracer remains sufficient for imaging purposes despite the degradation observed in serum. It is possible that the conjugate is more stable within the tumor microenvironment than in the circulating blood, while partial dissociation in the bloodstream may also contribute to the relatively rapid clearance of [^52^Mn]Mn-BPPA-bevacizumab from the blood pool. Future studies employing alternative linker chemistries will be necessary to further clarify this mechanism and to improve the overall radiochemical purity and stability of the radiolabeled construct.

From a translational perspective, the markedly improved tumor-to-background ratios achieved with [^52^Mn]Mn-BPPA-bevacizumab expand the feasible imaging window for VEGF-A-targeted immuno-PET and may enable more reliable lesion detection and quantitative assessment at later time points. Clinically, these results support the potential role of VEGF-A-targeted immuno-PET in the management of angiogenesis-driven tumors. A non-invasive method for quantifying VEGF-A expression could aid patient selection for bevacizumab therapy, provide early insights into therapeutic effectiveness, and enable longitudinal monitoring of treatment resistance. By combining the favorable physical properties of ^52^Mn with a highly stable chelation strategy, [^52^Mn]Mn-BPPA-bevacizumab represents a promising next-generation immuno-PET tracer for the evaluation of VEGF-A-expressing malignancies. Although the relatively long physical half-life of ^52^Mn (t_1/2_ = 5.6 days) represents an advantage for imaging slowly accumulating biomolecules such as monoclonal antibodies, it may also constitute practical limitations. Optimal tumor-to-background contrast with antibody-based tracers typically occurs several days after administration, which in the case of ^52^Mn-labeled antibodies may require imaging at late time points (e.g., day 4–7). Such delayed imaging protocols may complicate clinical logistics and reduce patient convenience compared with tracers that allow earlier imaging. In addition, the longer half-life of ^52^Mn may lead to higher radiation exposure relative to shorter-lived PET radionuclides, which could represent a limitation for clinical translation, particularly for imaging applications involving radiation-sensitive tissues such as the reproductive organs. Nevertheless, these characteristics are not unique to ^52^Mn, as several radionuclides commonly used for immuno-PET, including ^89^Zr (t_1/2_ = 3.27 days), similarly require delayed imaging to achieve optimal tumor contrast. Therefore, while the extended imaging window associated with ^52^Mn can be advantageous for antibody-based imaging, the practical implications of delayed imaging and radiation dosimetry should be considered when evaluating its clinical applicability.

### Limitations

A limitation of the present study is that the comparison between [^52^Mn]Mn-BPPA-bevacizumab and the previously reported [^52^Mn]Mn-DOTAGA-bevacizumab tracer was performed using data obtained in separate experiments. Although both studies employed the same tumor model, imaging system, acquisition protocol, and quantitative analysis methodology, inter-experimental variability cannot be fully excluded. Another limitation of the present study is the absence of ex vivo biodistribution measurements. Although PET/MRI imaging with attenuation correction allowed the non-invasive quantitative assessment of tracer kinetics and tumor-to-background ratios, ex vivo organ counting could have provided additional quantitative validation of the in vivo imaging results. Even though the pronounced tumor uptake observed in vivo suggests that the VEGF-A targeting capability of the bevacizumab remains functionally intact, a further limitation of this study is the absence of a dedicated in vitro binding or competitive blocking assay to directly confirm preservation of VEGF-A binding affinity following BPPA-Bn-pMMA conjugation.

## 4. Materials and Methods

### 4.1. Radiochemistry and Radiotracer Preparation

Analytical measurements were carried out with a UPLC-UV-RA-MS system which consists of an Acquity I-class UPLC (Waters, Milford, MA, USA) with a RA detector (Berthold, Bad Wildbad, Germany) and a G2 Q-TOF ESI MS (Waters, Milford, MA, USA). Analytical conditions: Column: Waters Protein xBridge SEC 4.6 × 150 mm and BioResolve SEC mAb Column, 7.8 × 300 mm; Eluents: 100 mM pH 7.2 NH_4_OAc isocratic and 0.1% HCOOH/ACN gradient separation.

#### 4.1.1. Conjugation of BPPA-Bevacizumab

Bevacizumab (Avastin^®^, Roche Pharma AG, Grenzach-Wyhlen, Germany, c = 25 mg/mL, M ~149,199 g/mol) was subjected to buffer exchange and concentration using Amicon Ultra 0.5 mL (30 kDa) centrifugal filter. Washing steps were performed three times with 400 µL of deionized water (11,000 rpm, 15 min, 18 °C). For the conjugation, the purified antibody (100 µL, 25 mg/mL) was diluted in 400 µL of 0.1 M NaHCO_3_ buffer (pH = 8.2). The synthesis of the BPPA chelator was performed as described previously [18]. The BPPA-Bu-pMMA bifunctional chelator was added in a 20-fold molar excess. The reaction mixture was incubated at room temperature for 24 h. The resulting conjugate was purified and transferred into a 0.1 M NaOAc buffer using Amicon Ultra filters (MilliporeSigma, Burlington, MA, USA), with the final protein concentration adjusted to 25 mg/mL.

#### 4.1.2. Radiolabeling with ^52^Mn

Manganese-52 was produced by proton irradiation of a natural chromium target using a 14 MeV beam via the ^52^Cr(p,n)^52^Mn nuclear reaction according to a previously reported procedure [33]. Radiochemical separation of ^52^Mn from the dissolved CrCl_3_ target material was performed, employing AG1-X8 anion-exchange resin with 3% (*v*/*v*) HCl in absolute ethanol and 0.1 M HCl as eluents. A subsequent purification step using DGA resin was applied to remove residual metallic impurities, including iron and copper.

The ^52^Mn isotope was utilized in a 0.1 M HCl solution. For the labeling procedure, 57 µL of ^52^Mn (12.8 MBq) was buffered with 61 µL of 0.1 M HEPES, and the pH was adjusted by adding 14 µL of 0.5 M NaOH. Subsequently, 12 µL of the BPPA-bevacizumab conjugate (0.3 mg of protein) was added to the solution. The reaction mixture was incubated at room temperature for 15 min. Radiochemical purity (RCP) was monitored using radio-thin layer chromatography (radio-TLC, 0.1 M citric acid as mobile phase) and radio-HPLC (Bioresolve antibody column), and the specific activity of the labeled conjugate was calculated.

#### 4.1.3. Lipophilicity Measurement

The lipophilicity of the ^52^Mn-BPPA-bevacizumab was determined using the shake-flask method to calculate the n-octanol/water partition coefficient (log P). Briefly, 10 µL of the radiolabeled conjugate was added to a mixture of 500 µL of n-octanol and 490 µL of water. The mixture was shaken at room temperature for 30 min. After phase separation, aliquots of 100 µL were collected from the organic phase, while 10 µL from the aqueous phase was diluted to 100 µL, and the radioactivity was measured using a gamma counter (Perkin Elmer, Shelton, CT, USA).

#### 4.1.4. Dose Preparation for In Vivo Studies

The radiolabeled product was divided into three equal aliquots (45 µL each) and diluted with 200 µL of 0.9% NaCl. Each administered dose was 3.8 MBq, corresponding to 0.01 mg of protein per injection.

### 4.2. Cell Line and Tumor Model

Human KB-3-1 cervix carcinoma cell line, known to overexpress VEGF-A [34], was used to establish the experimental tumor model. The KB-3-1 cell line was obtained from Dr. Katalin Goda (University of Debrecen, Faculty of Medicine, Department of Biophysics and Cell Biology).

Cell culturing conditions, maintenance, and preparation for inoculation were identical to those described previously [15]. Briefly, KB-3-1 cells were cultured in Dulbecco’s Modified Eagle Medium (DMEM, GIBCO Life Technologies Magyarország Ltd., Budapest, Hungary) supplemented with fetal bovine serum (10%, heat-inactivated FBS from GIBCO, Life Technologies Magyarország Ltd., Budapest, Hungary), antibiotics and antimycotics (1%, Sigma-Aldrich, Merck KGaA, Darmstadt, Germany), and maintained under standard conditions (37 °C, 5% CO_2_). Cell viability before inoculation was confirmed by trypan blue exclusion.

For tumor injection, 5 × 10^6^ KB-3-1 tumor cells were harvested and injected subcutaneously (150 µL 0.9% NaCl) into the shoulder region of the animals. Tumor growth was monitored regularly, and imaging experiments were initiated once tumors reached the predefined size range used in the reference study [15].

### 4.3. Animals

All animal experiments were performed in full compliance with national and European regulations governing the use of laboratory animals and were approved by the local institutional animal ethics committee (permission number: 16/2020/DEMÁB). Female CB17 SCID mice were housed under specific pathogen-free conditions with ad libitum access to food and water.

Animal strain, age, housing conditions, anesthesia protocols, and tumor inoculation procedures were identical to those described in our previously published cervix carcinoma immuno-PET study and are therefore not repeated here [15]. Sample size (*n* = 5 animals per time point) was chosen based on feasibility and in line with previously published immuno-PET studies using the same tumor model and imaging methodology. No a priori sample size calculation was performed, as the study was designed as an exploratory preclinical imaging investigation.

Humane endpoints were predefined prior to study initiation and included excessive tumor burden, significant weight loss, impaired mobility, or signs of pain or distress. Animals were monitored throughout the study period, and euthanasia would have been performed if humane endpoint criteria were met. No expected or unexpected adverse events occurred, and no animals required early termination.

### 4.4. In Vivo PET/MRI

In vivo PET/MRI was performed using a dedicated preclinical *nanoScan* PET/MRI system (Mediso Ltd., Budapest, Hungary) identical to those described in our previously published study [15]. Animals were anesthetized with isoflurane and injected intravenously via the tail vein with [^52^Mn]Mn-BPPA or [^52^Mn]Mn-BPPA-bevacizumab.

Whole-body PET scans were acquired at multiple time points post-injection (4 h, 24 h, 48 h, 72 h, 120 h, 168 h, and 240 h). MRI scans were performed for anatomical co-registration and accurate localization of tracer uptake. Three-dimensional GRE EXT multi-FOV MRI was performed using the following parameters: TR/TE = 15/2 ms, phase: 100, field of view: 60 mm, and NEX = 2. PET image reconstruction was carried out using a 3D iterative algorithm with appropriate corrections for attenuation, decay, and scatter, as detailed in our previous study [15].

### 4.5. Image Analysis and Quantification

Reconstructed PET/MRI datasets were analyzed using dedicated image analysis software (InterView™ FUSION v3.10, Mediso Ltd., Budapest, Hungary), in the same way as detailed in our previous study [15]. Volumes of interest (VOIs) of 3 mm diameter were manually delineated over tumors and major organs, including liver, kidneys, spleen, lungs, muscle, blood pool, pancreas, joint, and salivary gland, based on anatomical MRI guidance. Quantitative image analysis was conducted without knowledge of time point.

Tracer uptake was quantified using the mean standardized uptake values (SUV_mean_). Tumor-to-background ratios were calculated by dividing tumor SUV_mean_ values by corresponding SUV_mean_ values of selected reference organs. Time–activity curves were generated to evaluate tracer kinetics and retention patterns.

For comparison, tumor-to-background ratios obtained with [^52^Mn]Mn-BPPA-bevacizumab were directly compared with previously published values derived from [^52^Mn]Mn-DOTAGA-bevacizumab, acquired under identical experimental and analytical conditions [15].

### 4.6. Immunohistochemistry

Five micrometer thick frozen tumor sections were fixed in acetone (at −20 °C for 10 min). Sections were then washed and incubated with 0.3% H_2_O_2_ in methanol for 20 min to quench endogenous peroxidase activity. For blocking the nonspecific binding, 1% BSA (20 min) was used, then sections were incubated with rabbit anti-VEGF monoclonal antibody (at room temperature for 60 min in 1:200 dilution; Merck KGaA, Darmstadt, Germany). After washing with PBS, anti-mouse EnVision Detection Systems, Peroxidase/DAB (K500711-2; Agilent-Dako, Santa Clara, CA, USA) was used to visualize the primary antibodies, and sections were counterstained with hematoxylin. Negative controls were obtained by omitting the primary antibody.

### 4.7. Statistical Analysis

For comparative purposes, data obtained with [^52^Mn]MnCl_2_ and [^52^Mn]Mn-DOTAGA-bevacizumab were taken from our previously published study using the same tumor model and imaging protocol [15].

Quantitative data are presented as mean ± standard deviation (SD). Statistical analysis was performed using two-way ANOVA with Šídák’s post hoc test. Differences between groups were considered statistically significant at *p* < 0.05. MedCalc software v18.5 (Mariakerke, Belgium) and GraphPad Prism 10 (La Jolla, CA, USA) were used.

### 4.8. Use of GenAI

Generative artificial intelligence (GenAI) was used to aid proper English wording and phrasing. ChatGPT 5.2 and Grammarly v9.96 were used for this purpose. The authors have reviewed and edited the output and take full responsibility for the content of this publication. GenAI was not used for other purposes like generating graphics, or to assist in study design, data collection, analysis, or interpretation.

## 5. Conclusions

This study demonstrates that [^52^Mn]Mn-BPPA-bevacizumab exhibits sustained VEGF-A-specific tumor uptake, and significantly improved tumor-to-background ratios compared with the DOTAGA-based analogue. The enhanced imaging contrast highlights the importance of optimized chelation for ^52^Mn-based immuno-PET tracers. These favorable pharmacokinetic and imaging properties support the potential of [^52^Mn]Mn-BPPA-bevacizumab for non-invasive assessment of angiogenesis in cervical cancer. Further translational studies are warranted to evaluate its clinical applicability.

## Data Availability

The original contributions presented in this study are included in the article and Supplementary Materials. Further inquiries can be directed to the corresponding author.

## Supplementary Materials

The following supporting information can be downloaded at: https://www.mdpi.com/article/10.3390/ph19030517/s1, Figure S1: Mass spectrometry characterization of [^52^Mn]Mn-BPPA-bevacizumab; Figure S2: Serum stability of [^52^Mn]Mn-BPPA-bevacizumab obtained at 20 min, 220 min, 1 day, and 4 days after incubation in serum; Table S1: [^52^Mn]Mn-BPPA biodistribution expressed in SUV_mean_; Table S2: Biodistribution and tumor uptake of [^52^Mn]Mn-BPPA-bevacizumab expressed in SUV_mean_.

## Abbreviations

The following abbreviations are used in this manuscript:

ANOVA	Analysis of Variance
BPPA	Bis-Pyclen Picolinate
BSA	Bovine Serum Albumin
DAB	3,3′-Diaminobenzidine
DMEM	Dulbecco’s Modified Eagle Medium
DOTAGA	2,2′,2″-(10-(1-carboxy-4-((4-isothiocyanatobenzyl)amino)-4-oxobutyl)-1,4,7,10-tetraazacyclododecane-1,4,7-triyl)triacetic acid
FBS	Fetal Bovine Serum
FDA	Food and Drug Administration
H_2_O_2_	Hydrogen Peroxide
HCl	Hydrochloric Acid
HEPES	4-(2-Hydroxyethyl)-1-Piperazineethanesulfonic Acid
HPLC	High-Performance Liquid Chromatography
IHC	Immunohistochemistry
^52^Mn	Manganese-52
MRI	Magnetic Resonance Imaging
MS	Mass Spectrometry
NaCl	Sodium Chloride
PBS	Phosphate-Buffered Saline
PET	Positron Emission Tomography
RCP	Radiochemical Purity
SD	Standard Deviation
SUV_mean_	Mean Standardized Uptake Value
TLC	Thin-Layer Chromatography
VEGF-A	Vascular Endothelial Growth Factor A
VEGFR	Vascular Endothelial Growth Factor Receptor
VOI	Volume of Interest
^89^Zr	Zirconium-89
