# Supplementary material for: Enhanced Tumor-to-Background Contrast with [52Mn]Mn-BPPA-Bevacizumab VEGF-Targeted Immuno-PET in Cervical Cancer
Source: Pharmaceuticals (Basel). 2026 Mar 22;19(3):517. doi: 10.3390/ph19030517 (PMC13028754; doi:10.3390/ph19030517)
Supplement: Supplementary file 1 [file pharmaceuticals-19-00517-s001.zip › pharmaceuticals-4185403-supplementary.pdf]

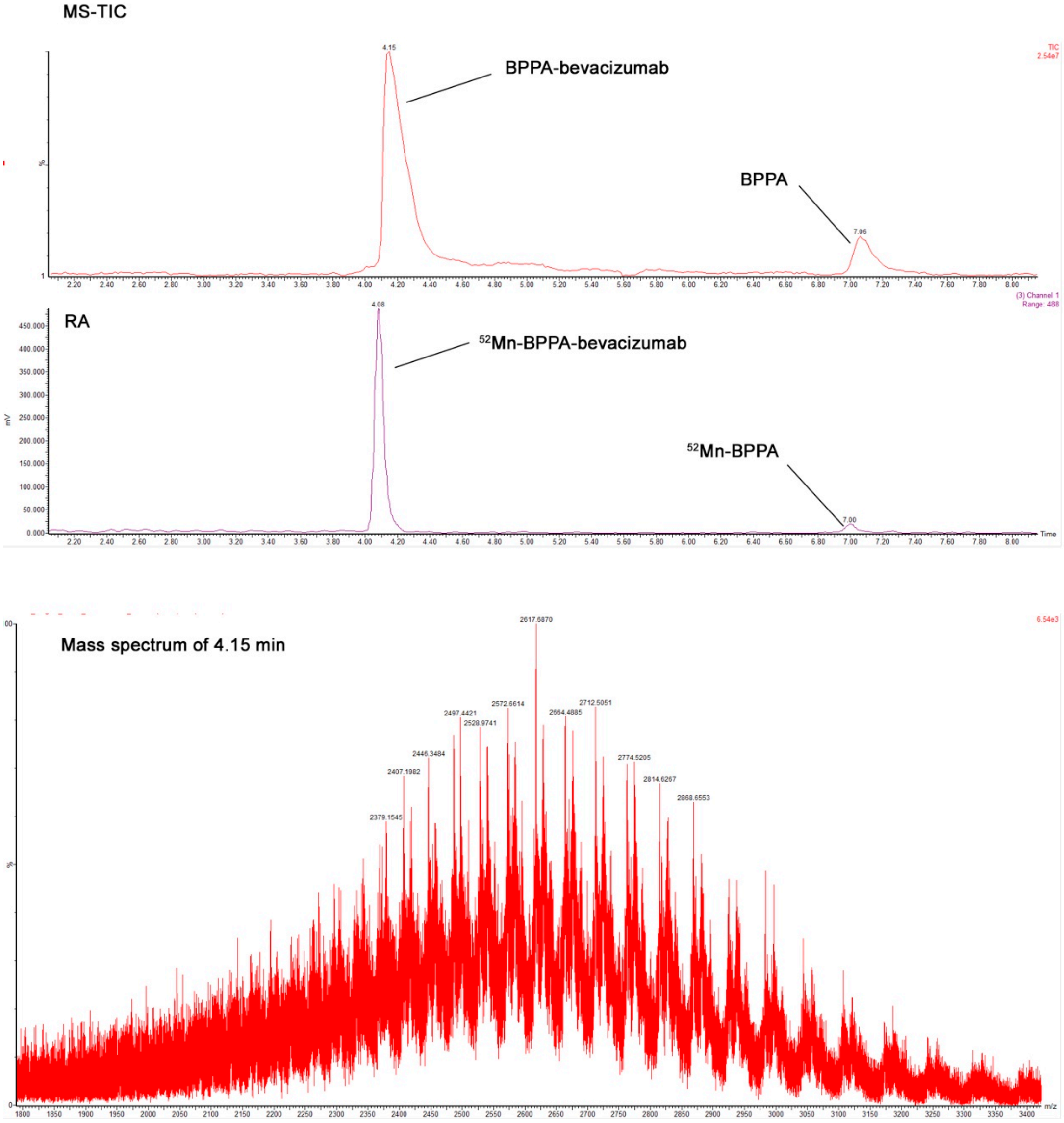

Figure S1. Mass spectrometry characterization of [^{52}Mn]Mn-BPPA-bevacizumab.

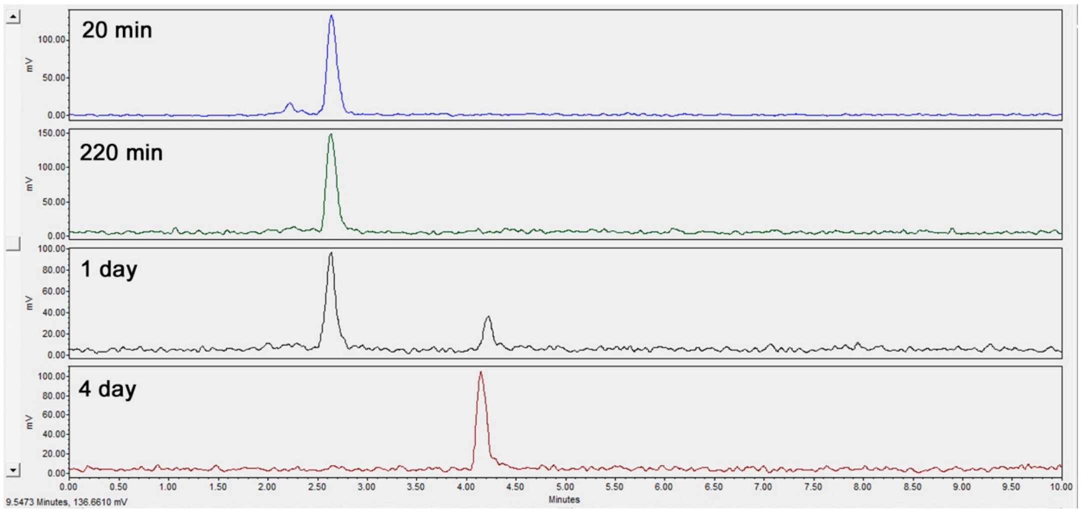

Figure S2. Serum stability of [^{52}Mn]Mn-BPPA-bevacizumab obtained at 20 minutes, 220 minutes, 1 day, and 4 days after incubation in serum.

Table S1. [52Mn]Mn-BPPA biodistribution expressed in SUV_{mean}.

	Blood	
**time (h)**	**Mean**	**SD**
4	0.02	0.00
24	0.01	0.00
48	0.01	0.00
72	0.01	0.00
120	0.01	0.00
168	0.01	0.00
240	0.01	0.00
	**Liver**	
**time (h)**	**Mean**	**SD**
4	0.35	0.06
24	0.16	0.03
48	0.10	0.01
72	0.09	0.01
120	0.06	0.01
168	0.05	0.01
240	0.03	0.01
	**Kidney**	
**time (h)**	**Mean**	**SD**
4	0.13	0.05
24	0.23	0.05
48	0.15	0.01
72	0.14	0.03
120	0.12	0.02
168	0.08	0.02
240	0.05	0.01
	**Spleen**	
**time (h)**	**Mean**	**SD**
4	0.06	0.02
24	0.04	0.03
48	0.03	0.01
72	0.03	0.01
120	0.02	0.01
168	0.02	0.02
240	0.02	0.02
	**Pancreas**	
**time (h)**	**Mean**	**SD**
4	0.13	0.02
24	0.11	0.03
48	0.16	0.02
72	0.13	0.01
120	0.14	0.03
168	0.13	0.02
240	0.09	0.02
	**Salivary gland**	
**time (h)**	**Mean**	**SD**
4	0.02	0.01
24	0.03	0.01
48	0.05	0.01
72	0.05	0.01
120	0.05	0.01
168	0.05	0.01
240	0.04	0.02
	**Muscle**	
**time (h)**	**Mean**	**SD**
4	0.01	0.01
24	0.00	0.01
48	0.01	0.01
72	0.02	0.01
120	0.01	0.01
168	0.01	0.01
240	0.00	0.01
	**Joint**	
**time (h)**	**Mean**	**SD**
4	0.01	0.01
24	0.02	0.01
48	0.01	0.01
72	0.02	0.01
120	0.02	0.00
168	0.01	0.00
240	0.01	0.01
	**Lung**	
**time (h)**	**Mean**	**SD**
4	0.02	0.01
24	0.01	0.01
48	0.01	0.00
72	0.01	0.01
120	0.01	0.01
168	0.00	0.00
240	0.00	0.00

Table S2. Biodistribution and tumor uptake of [^{52}Mn]Mn-BPPA-bevacizumab expressed in SUV_{mean}.

	**Blood**	
**time (h)**	Mean	SD
**4**	2.45	0.45
**24**	1.32	0.10
**48**	0.97	0.07
**72**	0.79	0.06
**120**	0.46	0.01
**168**	0.29	0.01
**240**	0.17	0.01
	**Liver**	
**time (h)**	Mean	SD
**4**	3.25	0.11
**24**	2.23	0.27
**48**	1.64	0.19
**72**	1.10	0.13
**120**	0.78	0.08
**168**	0.46	0.04
**240**	0.30	0.06
	**Kidney**	
**time (h)**	Mean	SD
**4**	1.43	0.21
**24**	1.32	0.22
**48**	1.26	0.23
**72**	1.31	0.34
**120**	1.42	0.41
**168**	1.36	0.48
**240**	1.29	0.37
	**Spleen**	
**time (h)**	Mean	SD
**4**	2.69	0.01
**24**	1.67	0.13
**48**	1.45	0.16
**72**	1.05	0.14
**120**	0.90	0.08
**168**	0.75	0.08
**240**	0.45	0.23
	**Pancreas**	
**time (h)**	Mean	SD
**4**	1.03	0.12
**24**	0.93	0.19
**48**	0.89	0.17
**72**	0.94	0.24
**120**	0.97	0.16
**168**	0.90	0.05
**240**	0.78	0.14
	**Salivary gland**	
**time (h)**	Mean	SD
**4**	0.34	0.06
**24**	0.53	0.06
**48**	0.45	0.10
**72**	0.54	0.13
**120**	0.54	0.06
**168**	0.60	0.09
**240**	0.56	0.08
	**Lung**	
**time (h)**	Mean	SD
**4**	1.25	0.23
**24**	0.73	0.02
**48**	0.60	0.03
**72**	0.46	0.03
**120**	0.29	0.05
**168**	0.22	0.02
**240**	0.12	0.03
	**Muscle**	
**time (h)**	Mean	SD
**4**	0.20	0.02
**24**	0.21	0.02
**48**	0.19	0.02
**72**	0.17	0.02
**120**	0.14	0.03
**168**	0.12	0.02
**240**	0.10	0.02
	**Joint**	
**time (h)**	Mean	SD
**4**	0.58	0.11
**24**	0.47	0.01
**48**	0.49	0.01
**72**	0.35	0.05
**120**	0.32	0.03
**168**	0.24	0.03
**240**	0.20	0.07
	**Tumor**	
**time (h)**	Mean	SD
**4**	1.07	0.57
**24**	1.64	0.25
**48**	2.06	0.25
**72**	2.42	0.16
**120**	2.38	0.13
**168**	2.35	0.21
**240**	1.77	0.24
